# Pertussis-Associated Myocarditis in an Adolescent: A Case Report

**DOI:** 10.7759/cureus.95226

**Published:** 2025-10-23

**Authors:** Noora AlSuwaidi, Bosaina Otour, Amer Salman

**Affiliations:** 1 Pediatric Emergency Medicine, Al Jalila Children's Specialty Hospital, Dubai, ARE

**Keywords:** cardiac, infection, myocarditis, pertussis, rash

## Abstract

Myocarditis is an uncommon complication of infectious diseases in children, most often viral in origin. Bordetella (B.) pertussis infection rarely causes myocardial involvement, with very few cases reported in the literature and even fewer in adolescents. We describe a rare case of acute myocarditis in a previously healthy 16-year-old male with confirmed B. pertussis infection, who presented atypically with abdominal pain and rash in the absence of a characteristic pertussive cough. Review of prior reports suggests pertussis myocarditis in adolescents is exceptionally rare, with variable presentations but generally favorable outcomes. While myocarditis has also been reported extremely rarely following pertussis-containing vaccines, no causal relationship has been established. This case highlights the importance of considering pertussis in the differential diagnosis of myocarditis, even when respiratory features are absent.

## Introduction

Pertussis, a respiratory infection caused by Bordetella pertussis, persists as a global health concern even with widespread immunization programs [1]. While classic symptoms include paroxysmal cough, inspiratory whoop, and post-tussive vomiting, these features may not always be present in adolescents and adults, leading to diagnostic challenges [2]. Severe complications predominantly occur in infants, with cardiac involvement being particularly uncommon across all age groups [3]. Previous literature mainly documents cases of pertussis-associated myocarditis in neonates and young infants, often resulting in poor clinical outcomes such as cardiogenic shock and dilated cardiomyopathy. Adolescents with myocarditis related to pertussis infection are scarcely reported, often only as rare consequences of vaccination, with insufficient evidence linking causality. Proposed mechanisms underlying pertussis-associated myocarditis include direct myocardial injury from toxins produced by Bordetella pertussis, immune-mediated myocardial inflammation, and microvascular coronary dysfunction leading to perfusion abnormalities [4-6]. This case report highlights an atypical presentation of acute myocarditis in an adolescent with confirmed pertussis infection, manifesting as abdominal pain and rash, thereby broadening the known clinical spectrum.

## Case presentation

A previously healthy, 16-year-old male presented to the emergency department with a 1-day history of lower abdominal pain, chest pain, and shortness of breath. On the same day, he developed a generalized pruritic rash and a tactile fever. Two days earlier, he had experienced a sore throat and two episodes of non-bilious vomiting. Notably, there was no history of cough prior to or during hospitalization [2]. Upon examination, the patient was afebrile with a temperature of 36.5 °C, a respiratory rate of 18/min, oxygen saturation of 100% on room air, and a blood pressure of 118/75 mmHg. Both cardiovascular and respiratory examinations were unremarkable. Abdominal examination revealed right iliac fossa tenderness without signs of peritonism. A faint erythematous maculopapular rash was observed scattered across his trunk and upper limbs. Laboratory investigations revealed (Table 1).

**Table 1:** Lab investigation WBC = white blood cells; CRP = C-reactive protein; ng/mL = nanograms per milliliter; ng/L= nanograms per liter; g/dL= grams per deciliter; x10⁹/L= cells x 10⁹ per liter

Parameter	Result	Reference Range
WBC	8.2 × 10⁹/L	4.0–11.0 × 10⁹/L
Lymphocytes	3.26 × 10⁹/L	1.0–4.0 × 10⁹/L
Hemoglobin	15.9 g/dL	12.0–17.5 g/dL
Platelets	261 × 10⁹/L	150–400 × 10⁹/L
CRP	33.7 mg/L	0–5 mg/L
Troponin I	3.16 ng/mL	<0.04 ng/mL
Troponin T	233 ng/L	<14 ng/L

Imaging studies showed widespread ST elevation on the initial ECG. Abdominal ultrasound was inconclusive due to poor visualization of the appendix and showed no evidence of lymphadenitis or free fluid. Echocardiography revealed moderate left ventricular dysfunction with an ejection fraction (EF) between 41% and 46%. CT coronary angiography demonstrated limited right coronary artery perfusion with suboptimal opacification but did not reveal structural anomalies. Respiratory PCR detected Bordetella pertussis (cycle threshold: 28); other respiratory pathogens were negative. Blood cultures remained sterile. Initial management focused on suspected appendicitis, but the patient was transferred to cardiology after laboratory and imaging findings revealed myocarditis. Azithromycin 500 mg orally daily for five days was given as per the pertussis protocol. No inotropes, angiotensin-converting enzyme (ACE) inhibitors, or beta-blockers were required during hospitalization. Serial echocardiograms showed progressive improvement in left ventricular function, with EF rising to 55% at two weeks and normalizing to 62% at six weeks. At the two-month outpatient follow-up, he remained asymptomatic with no complications [3,7].

**Figure 1:**
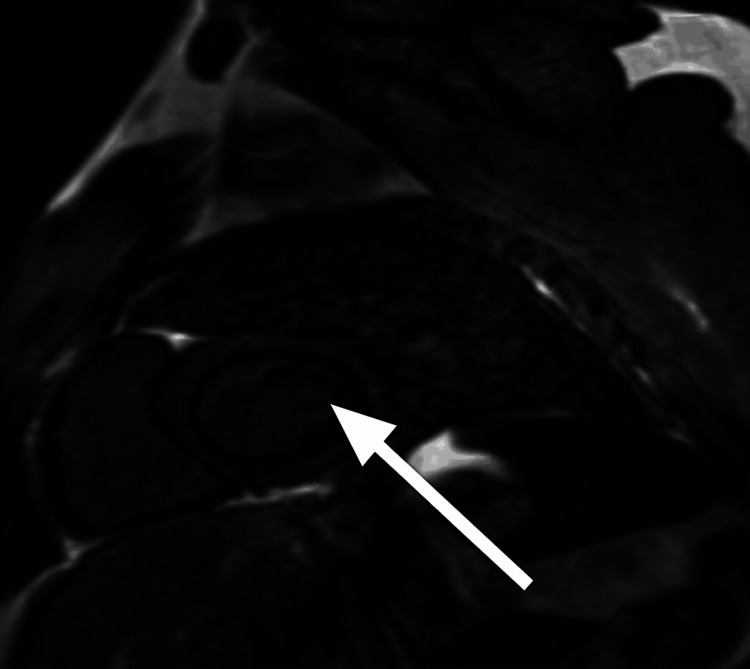
Cardiac magnetic resonance imaging (MRI) Short-axis view showing the left ventricle with myocardial involvement (arrow).

**Figure 2:**
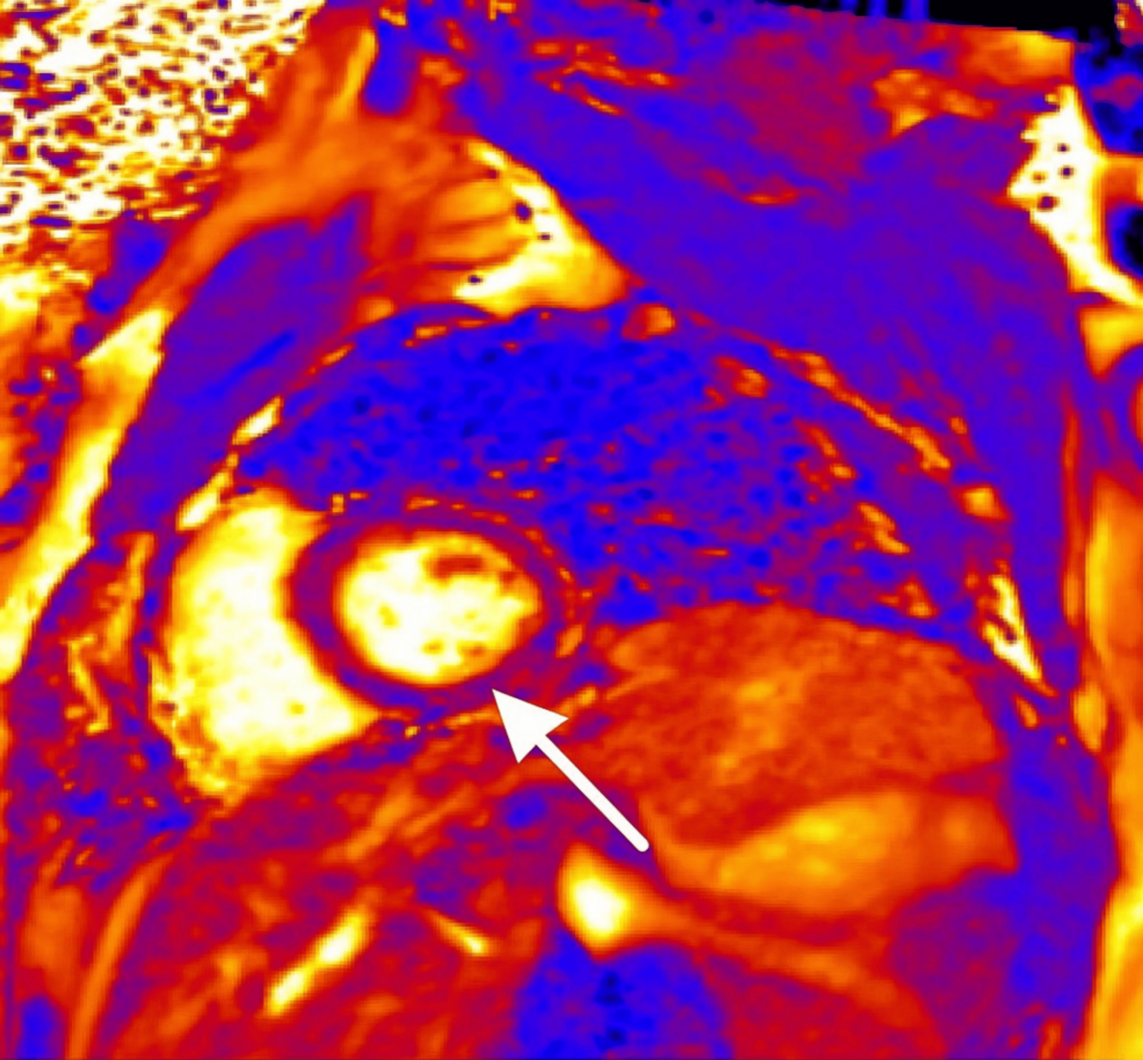
Cardiac magnetic resonance imaging (MRI), delayed TRUFI PSIR, short-axis view Demonstrating late gadolinium enhancement with epicardial involvement in the inferolateral midventricle, consistent with myocarditis (arrow). TRUFI = true fast imaging with steady-state free precession; PSIR = phase-sensitive inversion recovery

**Figure 3:**
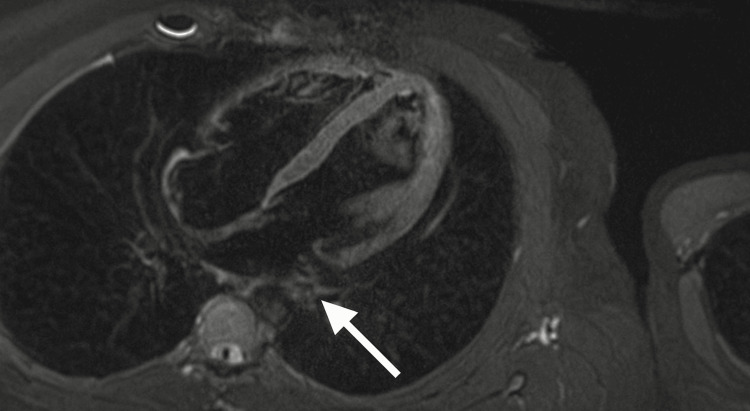
Cardiac magnetic resonance imaging (MRI), T2-weighted four-chamber view Useful for assessing myocardial edema. No active myocardial edema is seen, supporting the absence of acute inflammation (arrow).

Representative cardiac MRI slices are shown in Figures 1-3.

## Discussion

Myocarditis in childhood is most often triggered by viral agents, with bacterial etiologies representing a minority [1]. Bordetella pertussis-related myocarditis has been documented mainly in neonates and infants; adolescent cases are exceedingly rare and, when described, tend to be temporally associated with vaccination rather than confirmed infection [7]. This report is unique, as it documents acute myocarditis of clear pertussis etiology in an adolescent, with an atypical clinical presentation. Absence of cough in this patient is notable since it deviates from classic pertussis presentations. Hypotheses for this atypical manifestation include partial vaccine-derived immunity masking upper respiratory symptoms, systemic immune activation predominating over airway inflammation, or a greater tendency toward cardiac involvement in older children. The abdominal pain could plausibly stem from diaphragmatic irritation, mesenteric lymphadenitis secondary to pertussis, or hypoperfusion related to LV dysfunction. Pruritic rash, seldom described in previous reports, likely reflects immune-mediated systemic inflammation [2,6]. Diagnosis was supported by elevated cardiac biomarkers, confirmatory echocardiographic and CT findings, and PCR identification of the pathogen. Cardiac MRI, the gold standard for diagnosing myocarditis, was unfortunately unavailable, representing a limitation [6]. Nevertheless, clinical, laboratory, and molecular data provided a robust basis for pertussis-related myocarditis. Therapeutically, most adolescent patients recover with antimicrobial therapy and supportive management. Although myocarditis cases following pertussis vaccination are reported, their rarity and lack of mechanistic evidence argue against a causal link, emphasizing infection as the higher risk [8,9]. Vaccination remains pivotal for protection against severe sequelae such as myocarditis [1]. Recent literature continues to support recovery and atypical presentation in adolescent cases [7].

## Conclusions

Pertussis-associated myocarditis in adolescents is exceedingly rare. This case is notable for its atypical presentation--abdominal pain and rash without cough--which delayed recognition. Clinicians should maintain a broad differential when assessing adolescents with chest pain, elevated cardiac markers, and unusual features. Early cardiac evaluation and pathogen identification guide management. While myocarditis is sporadically reported post-vaccination, overwhelming evidence supports vaccination for the prevention of severe pertussis-related morbidity.
